# ARMv8-A System Semantics: Instruction Fetch in Relaxed Architectures

**DOI:** 10.1007/978-3-030-44914-8_23

**Published:** 2020-04-18

**Authors:** Ben Simner, Shaked Flur, Christopher Pulte, Alasdair Armstrong, Jean Pichon-Pharabod, Luc Maranget, Peter Sewell

**Affiliations:** ETH Zurich, Zurich, Switzerland; 9 University of Cambridge, Cambridge, UK; 10 Inria Paris, Paris, France

## Abstract

Computing relies on *architecture specifications* to decouple hardware and software development. Historically these have been prose documents, with all the problems that entails, but research over the last ten years has developed rigorous and executable-as-test-oracle specifications of mainstream architecture instruction sets and “user-mode” concurrency, clarifying architectures and bringing them into the scope of programming-language semantics and verification. However, the *system semantics*, of instruction-fetch and cache maintenance, exceptions and interrupts, and address translation, remains obscure, leaving us without a solid foundation for verification of security-critical systems software.

In this paper we establish a robust model for one aspect of system semantics: instruction fetch and cache maintenance for ARMv8-A. Systems code relies on executing instructions that were written by data writes, e.g. in program loading, dynamic linking, JIT compilation, debugging, and OS configuration, but hardware implementations are often highly optimised, e.g. with instruction caches, linefill buffers, out-of-order fetching, branch prediction, and instruction prefetching, which can affect programmer-observable behaviour. It is essential, both for programming and verification, to abstract from such microarchitectural details as much as possible, but no more. We explore the key architecture design questions with a series of examples, discussed in detail with senior Arm staff; capture the architectural intent in operational and axiomatic semantic models, extending previous work on “user-mode” concurrency; make these models executable as test oracles for small examples; and experimentally validate them against hardware behaviour (finding a bug in one hardware device). We thereby bring these subtle issues into the mathematical domain, clarifying the architecture and enabling future work on system software verification.

### 1 Introduction

Computing relies on the *architectural abstraction*: the specification of an envelope of allowed hardware behaviour that hardware implementations should lie within, and that software should assume. These interfaces, defined by hardware vendors and relatively stable over time, notionally decouple hardware and software development; they are also, in principle, the foundation for software verification. In practice, however, industrial architectures have accumulated great complexity and subtlety: the ARMv8-A and Intel architecture reference manuals are now 7476 and 4922 pages [9,26], and hardware optimisations, including out-of-order and speculative execution, result in surprising and poorly-understood programmer-observable behaviour. Architecture specifications have historically also been entirely informal, describing these complex envelopes of allowed behaviour solely in prose and pseudocode. This is problematic in many ways: do not serve as clear documentation, with the inevitable ambiguity and incompleteness of informal prose leaving major questions unanswered; without a specification that is executable as a test oracle (that can decide whether some observed behaviour is allowed or not), hardware validation relies on test suites that must be manually curated; without an architecturally-complete emulator (that can exhibit all allowed behaviour), it is very hard for software developers to "program to the specification" – they rely on test-and-debug development, and can only test above the hardware implementation(s) they have; and without a mathematically rigorous semantics, formal verification of hardware or software is impossible.

Over the last 10 years, much has been done to put architecture specifications on a more rigorous footing, so that a single specification can serve all those purposes. There are three main problems, two of which are now largely solved.

The first is the instruction-set architecture (ISA): the specification of the sequential behaviour of individual instructions. This is chiefly a problem of scale: modern industrial architectures such as Arm or x86 have large instruction sets, and each instruction involves many details, including its behaviour at different privilege levels, virtual-to-physical address translation, and so on – a single Arm instruction might involve hundreds of auxiliary functions. Recent work by Reid et al. within Arm [40,41,42] transitioned their internal ISA description into a mechanised form, used both for documentation and testing, and with him we automatically translated this into publicly available Sail definitions and thence into theorem-prover definitions [11,10]. Other related work is in §7.

The second is the relaxed-memory concurrent behaviour of "user-mode" operations: memory writes and reads, and the mechanisms that architectures provide to enforce ordering and atomicity (dependencies, memory barriers, load-linked/store-conditional operations, etc.). In 2008, for ARMv7, IBM POWER, and x86, this was poorly understood, and the architects regarded even their own prose specifications as inscrutable. Now, following extensive work by many people [36,37,19,18,22,8,31,45,7,46,48,35,6,2,47,13,1], ARMv8-A has a well-defined and simplified model as part of its specification [9, B2.3], including a prose transcription of a mathematical model [15], and an equivalence proof between operational and axiomatic presentations [36,37]; RISC-V has adopted a similar model [52]; and IBM POWER and x86 have well-established de-facto-standard models. All of these are experimentally validated against hardware, and supported by tools for exhaustively running tests [17,4]. The combination of these models and the ISA semantics above is enough to let one reason about or model-check concurrent algorithms.

That leaves the third part of the problem: the "system" semantics, of instruction-fetch and cache maintenance, exceptions and interrupts, and address translation and TLB (translation lookaside buffer) maintenance. Just as for "user-mode" relaxed memory, these are all areas where microarchitectural optimisations can have surprising programmer-visible effects, especially in the concurrent context. The mechanisms are relied on by all code, but they are explicitly managed only by systems code, in just-in-time (JIT) compilers, dynamic loaders, operating-system (OS) kernels, and hypervisors. This is, of course, exactly the security-critical computing base, currently trusted but not trustworthy, that is especially in need of verification – which requires a precise and well-validated definition of the architectural abstraction. Previous work has scarcely touched on this: none of seL4 [27], CertiKOS [24,23], Komodo [16], or [25,12], address realistic architecture concurrency, and they use (at best) idealised models of the sequential systems architecture. The CakeML [51,28] and CompCert [29] verified compilers target only sequential user-mode ISA fragments.

In this paper we focus on one aspect of system semantics: instruction fetch and cache maintenance, for ARMv8-A. The ability to execute code that has previously been written to data memory is fundamental to computing: fine-grained self-modifying code is now rare, and (rightly) deprecated, but program loading, dynamic linking, JIT compilation, debugging, and OS configuration all rely on executing code from data writes. However, because these are relatively infrequent operations, hardware designers have been able to optimise by partially separating the instruction and data paths, e.g. with distinct instruction caching, which by default may not be coherent with data accesses. This can introduce programmer-visible behaviour analogous to that of user-mode relaxed-memory concurrency, and require specific additional synchronisation to correctly pick up code modifications. Exactly what these are is not entirely clear in the current ARMv8-A architecture text, just as pre-2018 user-mode concurrency was not.

Our main contribution is to clarify this situation, developing precise abstractions that bring the instruction-fetch part of ARMv8-A system behaviour into the domain of rigorous semantics. Arm have stated [private communication] that they intend to incorporate a version of this into their architecture. We aim thereby to enable future work on system software verification using the techniques of programming languages research: program analysis, model-checking, program logics, etc. We begin (§2) by recalling the informal architectural guarantees that Arm provide, and the ways in which real-world software systems such as Linux, JavaScript, and WebAssembly change instruction memory. Then:

(1) **We explore the fundamental phenomena and architecture design questions with a series of examples** (§3). We explore the interactions between instruction fetching, cache maintenance and the 'usual' relaxed memory stores and loads, showing that instruction fetches are more relaxed, and how even fundamental coherence guarantees for data memory do not apply to instruction fetches. Most of these questions arose during the development of our models, in detailed ongoing discussion with the Arm Chief Architect and other Arm staff. They include questions of several different kinds. Six are clear from the Arm prose specification. Of the others: two are not implied by the prose but are natural choices; five involved substantive new choices by Arm that had not previously been considered and/or documented; for two, either choice could be reasonable, and Arm chose the simpler (and weaker) option; and for one, Arm were independently already strengthening the architecture to accommodate existing software.

(2) **We give an operational semantics for Arm instruction fetch and icache maintenance** (§4). This is in an abstract-microarchitectural style that supports an operational intuition for how hardware actually works, while abstracting from the mass of detail and the microarchitectural variation of actual hardware implementations. We do so by extending the Flat model [37] with simple abstractions of instruction caches and the coherent data cache network, in a way that captures the architectural intent, defining the entire envelope of behaviours that implementations should be allowed to exhibit.

(3) **We give a more concise presentation of the model in an axiomatic style** (§5), extending the "user-mode" axiomatic model from previous work [37,36,15,9], and intended to be functionally equivalent. We discuss how this too matches the architectural intent.

(4) **We validate all this** in two ways: by the extensive discussion with Arm staff mentioned above, and by experimental testing of hardware behaviour, on a selection of ARMv8-A cores designed by multiple vendors (§6). We run tests on hardware with a mild extension of the Litmus tool [5,7]. We make the operational model executable as a test oracle by integrating it into the RMEM tool and its web interface [17], introducing optimisations that make it possible to exhaustively execute the examples. We make the axiomatic model executable as a test oracle with a new tool that takes litmus tests and uses a Sail [11] definition of a fragment of the ARMv8-A ISA to generate SMT problems for the model. We then compare hardware and the two models for the handwritten tests (modulo two tests not supported by the axiomatic checker), compare hardware and the operational model on a suite of 1456 tests, automatically generated with an extension of the diy tool [3], and check the operational and axiomatic models against sets of previous non-ifetch tests. In all this data our models are equivalent to each other and consistent with hardware observations, except for one case where our testing uncovered a hardware bug on a Qualcomm device.

Finally, we discuss other related work (§7) and conclude (§8). We do all this for ARMv8-A, but other relaxed architectures, e.g. IBM POWER and RISC-V, face similar issues; our tests and tooling should enable corresponding work there.

The models are too large to include or explain in full here, so we focus on explaining the motivating examples, the main intuition and style of the operational model, in a prose rendering of its executable mathematics, and the definition of the axiomatic model. Appendices provide additional examples, a complete prose description of the operational model, and additional explanation of the axiomatic model. The complete executable mathematics version, the web-interface tool for running it, and our test results are at `https://www.cl.cam.ac.uk/~pes20/iflat/`.

*Caveats and Limitations* Our executable models are integrated with a substantial fragment of the Sail ARMv8-A ISA (similar to that used for CakeML), but not yet with the full ISA model [11,40,41,42]; this is just a matter of additional engineering. We only handle the 64-bit AArch64 part of ARMv8-A, not AArch32. We do not handle the interaction between instruction fetch and mixed-size accesses, or other variants of the cache maintenance instructions, e.g. those used for interaction with DMA engines, and variants by set or way instead of by virtual address. Finally, the equivalence between our operational and axiomatic models is validated experimentally. A proof of this equivalence is essential in the long term, but would be a major work in itself: the complexity makes mechanisation essential, but the operational model (in all its scale and complexity) has not yet been subject to mechanised proof. Without instruction fetch, a non-mechanised proof was the main result of an entire PhD thesis [36], and we expect the addition of instruction fetch to require global changes to the argument.

### 2 Industry Practice and the Existing ARMv8-A Prose

Computer architecture relies on a host of sophisticated techniques, including buffering, caching, prediction, and pipelining, for performance. For the normal memory reads and writes of "user-mode" concurrency, the programmer-visible relaxed-memory effects largely arise from store buffering and from out-of-order and speculative pipeline behaviour, not from the cache hierarchy (though some IBM POWER phenomena do arise from the interconnect, and from late processing of cache invalidates). All major architectures provide a strong per-location guarantee of *coherence*: for each memory location, different threads cannot observe the writes to that location in different orders. This is implemented in hardware by coherent cache protocols, ensuring (roughly) that each cache line is writable by at most one hardware thread at a time, and by additional machinery restricting store buffer and pipeline behaviour. Then each architecture provides additional synchronisation mechanisms to let the programmer enforce ordering properties involving multiple locations.

At first sight, one might expect instruction fetches to act like other memory reads but, because writes to instruction memory are relatively rare, hardware designers have adopted different caching mechanisms. The Arm architecture carefully does not mandate exactly what these must be, to allow a wide range of possible hardware implementations, but, for example, a high-performance Arm processor might have per-core separate L1 instruction and data caches, above a unified per-core L2 cache and an L3 cache shared between cores. There may also be additional structures, e.g. per-core fetch queues, and caching of decoded micro-operations. This instruction caching is not necessarily coherent with data memory accesses: *"the architecture does not require the hardware to ensure coherency between instruction caches and memory"* [9, B2.4.4 (B2-114)]; instead, programmers must use explicit cache maintenance instructions. The documentation gives a particular sequence of these: *"If software requires coherency between instruction execution and memory, it must manage this coherency using Context*

*synchronization events and cache maintenance instructions. The following code sequence can be used to allow a processing element (PE) to execute code that the same PE has written."*

```
; Coherency example for data and instruction accesses [...]
; Enter this code with <Wt> containing a new 32-bit instruction,
; to be held in Cacheable space at a location pointed to by Xn.
STR Wt, [Xn]; Store new instruction
DC CVAU, Xn ; Clean data cache by virtual address (VA) to PoU
DSB ISH     ; Ensure visibility of the data cleaned from cache
IC IVAU, Xn ; Invalidate instruction cache by VA to PoU
DSB ISH     ; Ensure completion of the invalidations
ISB         ; Synchronize the fetched instruction stream
```

At first sight, this may be entirely mysterious. The remainder of the paper establishes precise semantics for each instruction, explaining why each is required, but as a rough intuition:

1. The `DC CVAU,Xn` cleans this core's data cache for address `Xn`, pushing the new write far enough down the hierarchy for an instruction fetch that misses in the instruction cache to be guaranteed to see the new value. This point is the *Point of Unification* (PoU) and is usually the point where the instruction and data caches become unified (L2 for most modern devices).
2. The `DSB ISH` waits for the clean to have happened before letting the later instructions execute (without this, the sequence itself can execute out-of-order, and the clean might not have pushed the write down far enough before the instruction cache is updated). The `ISH` makes this specific to the *Inner Shareable Domain*: the processor itself, not the system-on-chip. We do not model shareability domains in this paper, so this is equivalent to a `DSB SY`.
3. The `IC IVAU,Xn` invalidates any entry for that address in the instruction caches for all cores, forcing any future fetch to miss in the instruction cache, and instead read the new value from the data memory hierarchy; it also touches some fetch queue machinery.
4. The second `DSB ISH` ensures the invalidation completes.
5. The final `ISB` flushes this core's pipeline, forcing a re-fetch of all program-order-later instructions.

Some hardware implementations provide extra guarantees, rendering the `DC` or `IC` instructions unnecessary. Arm allow software to discover this in an architectural way, by reading the `CTR_EL0` register's `DIC` and `IDC` bits. Our modelling handles this, but for brevity we only discuss the weakest case, with `CTR_EL0.DIC=CTR_EL0.IDC=0`, that requires full cache maintenance.

Arm make clear that instructions can be prefetched (perhaps speculatively): *"How far ahead of the current point of execution instructions are fetched from is IMPLEMENTATION DEFINED. Such prefetching can be either a fixed or a dynamically varying number of instructions, and can follow any or all possible future execution paths. For all types of memory, the PE might have fetched the instructions from memory at any time since the last Context synchronization event on that PE."*

Concurrent modification and instruction fetch require the same sequence, with an ISB on each thread that executes the new instructions, and the rest of the sequence on the modifying thread [9, B2.2.5 (B2-94)]. Concurrent modification without synchronisation is restricted to particular instructions (B (branch), BL (branch-and-link), BRK (break), SMC, HVC, SVC (secure monitor, hypervisor, and supervisor calls), ISB, and NOP), otherwise there could be *constrained unpredictable behaviour*: *"any behavior that can be achieved by executing any sequence of instructions that can be executed from the same Exception level"*. Concurrent modification of conditional branches is allowed but can result in the old condition with the new target address or vice versa.

All this gives some guidance for programmers, but it leaves the exact semantics of instruction fetch and those cache maintenance instructions unclear, and in practice software typically does not use the above sequence verbatim. For example, it may synchronise a range of addresses at once, looping the DC and IC parts, or the final ISB may be subsumed by instruction synchronisation from exception entry or return. Linux has many places where it modifies code at runtime: in boot-time patching of *alternatives*, modifying kernel code to specialise it to the particular hardware being run on; when the kernel loads code (e.g. when the user calls dl_open); and in the ptrace system call, used e.g. by the GDB debugger to patch arbitrary instructions with breakpoints at runtime. In Google's *Chrome* web browser, its WebAssembly and JavaScript just-in-time (JIT) compilers are required to both write new code during execution and modify existing code at runtime. In JavaScript, this modification happens inside a single thread and so is quite straightforward. The WebAssembly case is more complex, as one thread is modifying the code of another. A software thread can also be moved (by the OS or hypervisor) from one hardware thread to another, perhaps while it is in the middle of some instruction cache maintenance. Moreover, for security reasoning, we have to be able to bound the possible behaviour of arbitrary code.

All this means that we cannot treat the above sequence as a whole, as an opaque black box. Instead, we need a precise semantics for each individual instruction, but the existing prose documentation does not provide that.

The problem we face is to give such a semantics, that correctly defines behaviour in arbitrary concurrent contexts, that captures the Arm architectural intent, that is strong enough for software, and that abstracts from the variety of hardware implementations (e.g. with differing cache structures) that the architecture intends to allow – but which programmers should not have to think about.

### 3 Instruction Fetch Phenomena and Examples

We now describe the main instruction-fetch phenomena and architecture design questions for ARMv8-A, illustrated by handwritten litmus tests, to guide the following model design.

#### 3.1 Instruction-Fetch Atomicity

The first point, as mentioned in §2, is that concurrent modification and fetch is only permitted if the original and modified instructions are in a particular set: various branches, supervisor/hypervisor/secure-monitor calls, the `ISB` instruction synchronisation barrier, and `NOP`. Otherwise, the architecture permits *constrained unpredictable* behaviour, meaning that the resulting machine state could be anything that would be reachable by arbitrary instructions at the same exception level. The following W+F test illustrates this.

W+F AArch64

| Initial state: `0:W0="SUB X0,X0,#1", 0:X1=l` | |
|---|---|
| **Thread 0** | **Thread 1** |
| `STR W0,[X1] // modify Thread 1 at l` | `l: ADD X0,X0,#1 // initial code` |
| Allowed: constrained-unpredictable final state | |

In this test Thread 0 performs a memory store (with the `STR` instruction) to the code that Thread 1 is executing; overwriting the `ADD X0,X0,#1` instruction with the 32-bit encoding of the `SUB X0,X0,#1` instruction. If the fetch were atomic, the outcome of this test would be the result of executing either the `ADD` or the `SUB` instruction, but, since at least one of those is not in the set of the 8 atomically-fetchable instructions given previously, Thread 1 has constrained-unpredictable behaviour and the final state is very loosely constrained. Note, however, that this is nonetheless much stronger than the C/C++ whole-program undefined behaviour in the presence of a data race: unlike C/C++, a hardware architecture has to define a useful envelope of behaviour for arbitrary code, to provide guarantees for the rest of the system when one user thread has a race.

**Conditional Branches** For conditional branches, the Arm architecture provides a specific non-single-copy-atomic fetch guarantee: the execution will be consistent with either the old or new target, and either the old or new condition. For example, this W+F+branches test can overwrite a `B.EQ g` with a `B.NE h`, and end up executing `B.NE g` or `B.EQ h` instead of one of those. Our future examples will only modify `NOP`s and unconditional branch instructions.

W+F+branches AArch64

| Initial state: `0:W0="B.NE h", 0:X1=l` | |
|---|---|
| **Thread 0** | **Thread 1** |
| `STR W0,[X1]` | `l: B.EQ g` |
| Allowed: execute `"B.NE g"` | |

#### 3.2 Coherence

Data writes and reads are coherent, in Arm and in other major architectures: in any execution, for each address, the reads of each hardware thread must see a subsequence of the total *coherence order* of all writes to that address. The plain-data CoRR test [46] illustrates one case of this: it is forbidden for a thread to read a new write of `x` and then the initial state for `x`. However, instruction fetches are not necessarily coherent: one instruction fetch may be inconsistent with a program-order-previous fetch, and the data and instruction streams can become out-of-sync with each other. We explore three kinds of coherence:

– Instruction-to-Instruction Coherence: whether fetches of the same location must observe writes to the same location coherently.
– Data-to-Instruction Coherence: whether fetches and then reads to the same location must observe writes to the same location coherently.
– Instruction-to-Data Coherence: whether reads and then fetches of the same location must observe writes to the same location coherently.

**Instruction-to-Instruction Coherence** Arm explicitly do not guarantee any consistency between fetches of the same location: fetching an instruction does not mean that a later fetch of that location will not see an older instruction [9, B2.4.4]. This is illustrated by CoFF, like CoRR but with fetches instead of reads.

CoFF AArch64

| Initial state: `0:W0="B l1", 0:X1=f` | | |
|---|---|---|
| **Thread 0** | **Thread 1** | **Common** |
| `STR W0,[X1] //a` | `BL f`<br>`MOV X0,X10`<br>`BL f`<br>`MOV X1,X10` | `f:  B l0`<br>`l1: MOV X10,#2`<br>`    RET`<br>`l0: MOV X10,#1`<br>`    RET` |
| Allowed: `1:X0=2, 1:X1=1` | | |

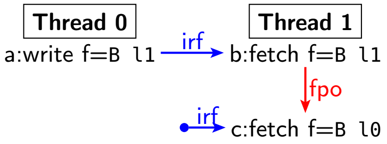

Here Thread 1 makes two calls to address `f` (`BL` is branch-and-link), while Thread 0 overwrites the instruction at that address. The interesting potential execution is that in which the first call to `f` fetches and executes the newly-written `B l1`, but the second call fetches and executes the original `B l0`. We can view such executions as graphs, similar to previous axiomatic-model candidate executions but with new fetch events, one per instruction, and new edges. As usual, we use `po` and `rf` edges for the program-order and reads-from relations, together with:

– `fe` (fetch-to-execute), which relates the fetch event of an instruction to all the execution events (memory writes, reads or barriers) of the instruction;
– `irf` (instruction-read-from), relating a write to all fetches that read from it (analogous to reads-from, `rf`); and
– `fpo` (fetch-program-order), relating fetches of instructions that are in program order (analogous to program order, `po`).

Edges from the initial state are drawn from a small circle. Since we do not modify the code of most locations, we usually omit the fetch events for those instructions, showing only a subgraph of the interesting events, e.g. as on the right above. For Arm, this execution is both architecturally allowed and experimentally observed.

Here, and in future tests, we assume some common code consisting of a function at address `f` which always has the same shape: a branch that might be overwritten, which selects a block that writes a value to register `X10` before returning. This is sometimes duplicated at different addresses (`f1`, `f2`, ...) or extended to `g`, with three cases. We sometimes elide the common code.

**Data-to-Instruction Coherence** Fetching from a particular write does imply that program-order-later reads from the same address will see that write (or a coherence successor thereof). This is a *data-to-instruction* coherence property, illustrated by CoFR below. Here Thread 1 fetches the newly-written `B l1` at `f` and then, when reading from `f` with its `LDR` load instruction, cannot read the original `B l0` instruction (it can only read the new `B l1`).

CoFR AArch64

| Initial state: `0:W0="B l1", 0:X1=f, 1:X2=f` | | |
|---|---|---|
| **Thread 0** | **Thread 1** | **Common** |
| `STR W0,[X1]` | `BL f`<br>`MOV X0,X10`<br>`LDR X1,[X2]` | `f: B l0`<br>`l1: MOV X10,#2`<br>`RET`<br>`l0: MOV X10,#1`<br>`RET` |
| Forbidden: `1:X0=2, 1:X1="B l0"` | | |

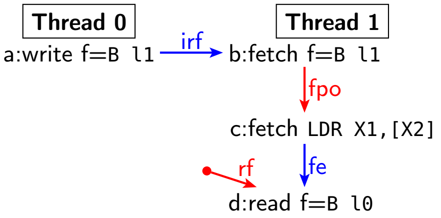

This is not clear in the existing prose specification, but the architectural intent that emerged during discussion with Arm is that the given execution should be forbidden, reflecting microarchitectural choices that (1) instructions decode in order, so the fetch `b` must occur before the read `d`, and (2) fetches that miss in the instruction cache must read from data storage, so the instruction cache cannot be ahead of the available data. This ensures that fetching from a write means that all threads are now guaranteed to read from that write (or another coherence-after it).

**Instruction-to-Data Coherence** In the other direction, reading from a particular write to some location does *not* imply that later fetches of that location will see that write (or a coherence successor), as in the following CoRF+ctrl-isb.

CoRF+ctrl-isb AArch64

| Initial state: `0:W0="B l1", 0:X1=f, 1:X2=f` | | |
|---|---|---|
| **Thread 0** | **Thread 1** | **Common** |
| `STR W0,[X1]` | `LDR X0,[X2]`<br>`CBNZ X0,l`<br>`l: **ISB**`<br>`BL f`<br>`MOV X1,X10` | `f: B l0`<br>`l1: MOV X10,#2`<br>`RET`<br>`l0: MOV X10,#1`<br>`RET` |
| Allowed: `1:X0="B l1", 1:X1=1` | | |

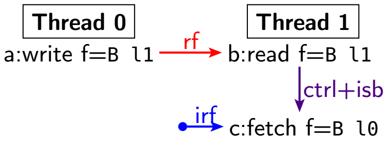

Here Thread 1 has a control dependency and an instruction synchronisation barrier (the `CBNZ` conditional branch, dependent on the value read by its `LDR` load, and `ISB`), abbreviated to ctrl+isb, between its load and the fetch from `f`. If the latter were a data load, this would ensure the two loads are satisfied in order. This is not explicit in the existing prose, but it is what one would expect, and it is observed in practice. Microarchitecturally, it is easily explained by an out-of-date entry for `f` in the instruction cache of Thread 1: if Thread 1 had previously fetched `f` (perhaps speculatively), and that instruction cache entry has not been evicted or explicitly invalidated since, then this fetch of `f` will simply read the old value from the instruction cache without going out to data memory. The `ISB` ensures that `f` is freshly fetched, but does not ensure that Thread 1's instruction cache is up-to-date with respect to data memory.

#### 3.3 Instruction Synchronisation

Instruction fetches satisfy few guarantees, so explicit synchronisation must be performed when modifying the instruction stream.

**Same-Thread Synchronisation** Test SM below shows the simplest self-modifying code case: without additional synchronisation, a write to program memory can be ignored by a program-order-later fetch.

SM AArch64

| Initial state: `0:W0="B l1", 0:X1=f` | |
|---|---|
| **Thread 0** | **Common** |
| `STR W0,[X1] // a`<br>`BL f`<br>`MOV X0,X10` | `f:  B l0`<br>`l1: MOV X10,#2`<br>`    RET`<br>`l0: MOV X10,#1`<br>`    RET` |
| Allowed: `1:X0=1` | |

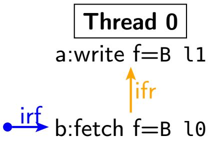

In this execution, the fetch `b`, fetching the instruction at `f`, fetches a value from a write coherence-before `a`, even though `b` is the fetch of an instruction program-order after `a`. We illustrate this with an *instruction from-reads* (`ifr`) edge. This is a derived relation, analogous to the usual *from-reads* (`fr`) relation, that relates each fetch to all writes that are coherence-after the write it read from; it is defined as $\texttt{ifr} = \texttt{irf}^{-1};\texttt{co}$. If the fetch were a data read, this would be a forbidden coherence shape (COWR). As it is, it is architecturally allowed, as described explicitly by Arm [9, B2.4.4], and it is experimentally observed on all devices we have tested. Microarchitecturally, this too is simply due to fetches from old instruction cache entries.

**Cache Maintenance** As we saw in §2, the Arm architecture provides cache maintenance instructions to synchronise the instruction and data streams: the `DC` data-cache clean and `IC` instruction-cache invalidate instructions. To forbid the relaxed outcome of SM, by forcing a fetch of the modified code, the specified sequence of cache maintenance instructions must be inserted, with an `ISB`.

SM+cachesync-isb AArch64

| Initial state: `0:W0="B l1", 0:X1=f` |
|---|
| **Thread 0** |
| `STR W0,[X1] //overwrite f with branch`<br>`DC CVAU,X1  //clean data cache`<br>`DSB ISH`<br>`IC IVAU,X1  //invalidate instruction cache`<br>`DSB ISH`<br>`ISB         //flush pipeline`<br>`BL f`<br>`MOV X0,X10` |
| Forbidden: `1:X0=1` |

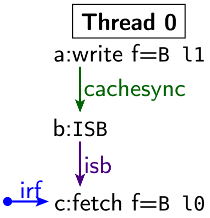

Now the outcome is forbidden. The cache synchronisation sequence `DC CVAU; DSB ISH; IC IVAU; DSB ISH` (which we abbreviate to a single `cachesync` edge) ensures that by the time the `ISB` executes, the instruction and data memory have been made coherent with each other for `f`. The `ISB` then ensures the final fetch of `f` is ordered after this sequence. The microarchitectural intuition for this was in §2; our §4 operational model will describe the semantics of each instruction.

**Cross-Thread Synchronisation** We now consider modifying code that can be fetched by other threads, using variants of the standard message-passing shape MP. That checks whether two writes (to different locations) on one thread can be seen out-of-order by two reads on another thread; here we replace one or both of those reads by fetches, and ask what synchronisation is required to ensure that the relaxed outcome is forbidden. Consider first an MP variant where the first write is of a new instruction, and the second is just a simple data memory flag:

MP.RF+dmb+ctrl-isb AArch64

| Initial state: `0:W0="B l1", 0:X1=f, 0:X2=1, 0:X3=x, 1:X2=x, [x]=0` | |
|---|---|
| **Thread 0** | **Thread 1** |
| `STR W0,[X1]`<br>`DMB ISH`<br>`STR X2,[X3]` | `LDR X0,[X2]`<br>`CBNZ X0,l`<br>`l: ISB`<br>`BL f`<br>`MOV X1,X10` |
| Allowed: `1:X0=1, 1:X1=1` | |

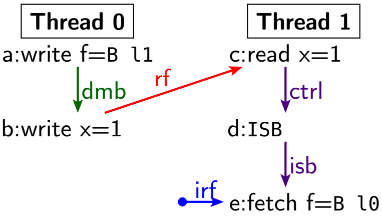

This test includes sufficient synchronisation on each thread to enforce thread-local ordering of data accesses: the `DMB` in Thread 0 ensures the writes `a` and `b` propagate to memory in program order, and the control-dependency into an `ISB` on Thread 1 ensures the read `c` and the fetch `e` happen in program order. However, as we saw in §2, this is not enough to synchronise concurrent modification and execution of code in ARMv8-A. Thread 0 needs the entire cache synchronization sequence (giving test MP.RF+cachesync+ctrl-isb, not shown), not just a `DMB`, to forbid this outcome.

Another variant of this MP-shape test where the message passing itself is done using modification of code gives a much stronger guarantee, as can be seen from the following MP.FR+dmb+fpo-fe test. This is not clear from the

MP.FR+dmb+fpo-fe AArch64

| Initial state: `0:X0=1, 0:X1=x, 1:X2=x, [x]=0, 0:W2="B l1", 0:X3=f` | |
|---|---|
| **Thread 0** | **Thread 1** |
| `STR X0,[X1]`<br>`DMB ISH`<br>`STR W2,[X3]` | `BL f`<br>`MOV X0,X10`<br>`LDR X1,[X2]` |
| Forbidden: `1:X0=2, 1:X1=0` | |

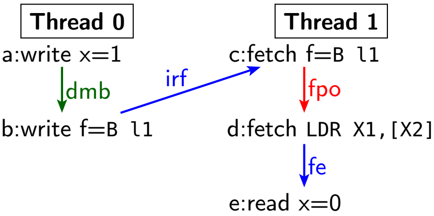

architecture manual, but this outcome is already forbidden with only the `DMB`.

This is for similar reasons to the above CoFR test: since Thread 1 fetched the updated value for `f`, we know that value must have reached at least the data caches (since that is where the instruction cache reads from) and therefore multi-copy atomicity guarantees that a normal load instruction will observe it.

The final variant of these MP-shaped tests has both Thread 0 writes be of new instructions. This idiom is very common in practice; it is currently how Chrome's WebAssembly JIT synchronises the modified thread with the new code.

MP.FF+dmb+fpo AArch64

| Initial state: `0:W0="B l1", 0:X1=f1, 0:W2="B l1", 0:X3=f2` | |
|---|---|
| **Thread 0** | **Thread 1** |
| `STR W0,[X1]`<br>`DMB ISH`<br>`STR W2,[X3]` | `BL f2`<br>`MOV X0,X10`<br>`BL f1`<br>`MOV X1,X10` |
| Allowed: `1:X0=2, 1:X1=1` | |

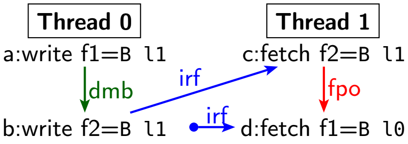

Without the full cachesync sequence on Thread 0, this is an allowed outcome. Interestingly, adding the cachesync sequence to Thread 0 (Test MP.FF+cachesync+fpo, not shown) is sufficient to make the outcome forbidden, without an `ISB` in Thread 1, as the cachesync sequence is intended to make it appear that fetches occur in program order. Microarchitecturally, that could be ensured in two ways: either by actually fetching in-order, or by making the `IC` instruction not only invalidate all the instruction caches (for this address) but also clean any core's pre-fetch buffer stale entries (for this address). Architecturally, this is not clear in the current prose, but, concurrent with this work, Arm were independently strengthening their definition to make it so.

**Incremental Synchronisation** The cache synchronisation sequence need not be contiguous, or even all in the same thread. So long as the sequence in its entirety has been performed by the time the fetch happens, then the instruction stream will have been made consistent with the data stream for that address.

This is demonstrated by the following test, where Thread 0 performs a write to `f` and then only a `DC` before synchronizing with Thread 1, which performs the `IC`, while Thread 2 observes the modified code. This can happen in practice when a software thread is migrated between hardware threads at runtime, by a hypervisor or OS. Thread 0 and Thread 1 may just represent the runtime scheduling of a single process, beginning execution on hardware Thread 0 but migrated to hardware Thread 1 between the `DC` and `IC` instructions. In the graph, the dcsync and icsync represent the `DC;DSB ISH` and `DSB ISH;IC;DSB ISH` combinations. The `DC` does not need a preceding `DSB ISH` because it is ordered w.r.t. the preceding store to the same cache line.

Here the `IC` gets broadcast to all threads [9, B2.2.5p3], and so the fact that it happens on a different thread to the `DC` does not affect the outcome. Similarly, if the `DC` were to happen on another thread first (to get the test MP.RF+[dc]-ic+ctrl-isb, not shown), then it would have the effect of ensuring consistency globally, for all threads.

ISA2.F+dc+ic+ctrl-isb AArch64

Initial state: `0:W0="B l1", 0:X1=f, 0:X2=1, 0:X3=x, [x]=0, 1:X4=f, 1:X1=x, 1:X2=1, 1:X3=y, [y]=0, 2:X2=y`

| Thread 0 | Thread 1 | Thread 2 |
|---|---|---|
| `STR W0,[X1]`<br>**`DC`** `CVAU, X1`<br>`DSB ISH`<br>`STR X2,[X3]` | `LDR X0,[X1]`<br>`DSB ISH`<br>**`IC`** `IVAU, X4`<br>`DSB ISH`<br>`STR X2,[X3]` | `LDR X0,[X2]`<br>`CBZ X0,l`<br>`l:`**`ISB`**<br>`BL f`<br>`MOV X1,X10` |

Forbidden: `1:X0=1, 1:X1=1`

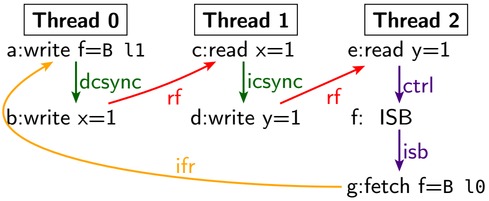

### 3.4 Multi-Copy Atomicity

For data accesses, the question of whether they are *multi-copy atomic* is a crucial one for relaxed architectures. IBM POWER, ARMv7, and pre-2018 ARMv8-A are/were non-multi-copy atomic: two writes to different addresses could become visible to distinct other threads in different orders. Post-2018 ARMv8-A and RISC-V are multi-copy atomic (or "other multi-copy-atomic" in Arm terminology) [37,36,9]: the programmer can assume there is a single shared memory, with all relaxed-memory effects due to thread-local out-of-order execution.

However, for fetches, due to the lack of any fetch atomicity guarantee for most instructions (§3.1), and the lack of coherent fetches for the others (§3.2), the question of multi-copy atomicity is not particularly interesting. Tests are either trivially forbidden (by data-to-instruction coherence) or are allowed but only the full cache synchronisation sequence provides enough guarantees to forbid it, and (§3.3) this ensures all cores will share the same consistent view of memory.

### 3.5 Strength of the `IC` Instruction

**Multiple Points of Unification** Cleaning the data cache, using the `DC` instruction, makes a write visible to instruction memory. It does this by pushing the write past the Point of Unification. However, there may be multiple Points of Unification: one for each core, where its own instruction and data memory become unified, and one for the entire system (or shareability domain) where all the caches unify. Fetching from a write implies that it has reached the closest PoU, but does not imply it has reached any others, even if the write originated from a distant core. Consider: Here Thread 0 modifies `f`, Thread 1 fetches the new value and performs just an `IC` and `DSB`, before signalling Thread 0 which also fetches `f`. That `IC` is not strong enough to ensure that the write is pulled into the instruction cache of Thread 0.

This is not clear in the existing prose, but the architectural intent is that it be allowed (i.e., that `IC` is weak in this respect). We have not so far observed it in practice. The write may have passed the Point of Unification for Thread 1, but not the shared Point of Unification for both threads. In other words, the write might reach Thread 1's instruction cache without being pushed down from Thread 0's data cache. Microarchitecturally this can be explained by *direct data*

SM.F+ic AArch64

| Initial state: `0:W0="B l1", 0:X4=f, 0:X3=x, [x]=0, 1:X4=f, 1:X2=1, 1:X3=x` | |
|---|---|
| **Thread 0** | **Thread 1** |
| `STR W0,[X4]`<br>`LDR X2,[X3]`<br>`CBZ X2,l`<br>`l: ISB`<br>`BL f`<br>`MOV X1,X10` | `BL f`<br>`MOV X0,X10`<br>`IC IVAU, X4`<br>`DSB ISH`<br>`STR X2,[X3]` |
| Allowed: `1:X0=2, 0:X2=1, 0:X1=1` | |

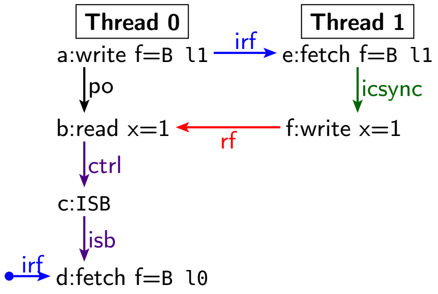

*intervention* (DDI), an optimisation allowing cache lines to be migrated directly from one thread's (data) cache to another. The line could be migrated from Thread 0 to Thread 1, then pushed past Thread 1's Point of Unification, making it visible to Thread 1's instruction memory without ever making it visible to Thread 0's own instruction memory. The lack of coherence between instruction and data caches would make this observable, even in multi-copy atomic machines.

**Stale Fetches** So far, we have only talked about fetching from two distinct writes. But theoretically there is no limit to how far back we can fetch from, with insufficient synchronization. The MP.RF+dmb+ctrl-isb test (§3.3) required the full `cachesync` sequence to forbid the given behaviour. Below we give a test, FOW, similar to that MP-shaped test but allowing many consumer threads to independently and simultaneously see different values in their instruction memory, even after invalidating their caches.

FOW AArch64

| Initial state: `0:W0="B l1", 0:X2=g, 0:W1="B l2", 0:X3=1, 0:X4=x, [x]=0, 1:X4=x, 2:X4=x` | | | |
|---|---|---|---|
| **Thread 0** | **Thread 1** | **Thread 2** | **Common** |
| `STR W0,[X2]`<br>`STR W1,[X2]`<br>`DSB ISH`<br>`IC IVAU, X2`<br>`DSB ISH`<br>`STR X3,[X4]` | `LDR X0, [X4]`<br>`CBNZ X0, la`<br>`la: ISB`<br>`BL g`<br>`MOV X1,X10` | `LDR X0, [X4]`<br>`CBNZ X0, lb`<br>`lb: ISB`<br>`BL g`<br>`MOV X1,X10` | `g: B l0`<br>`l2: MOV X10, #3`<br>`RET`<br>`l1: MOV X10, #2`<br>`RET`<br>`l0: MOV X10, #1`<br>`RET` |
| Allowed: `1:X0=1, 1:X1=2, 2:X0=1, 2:X1=1` | | | |

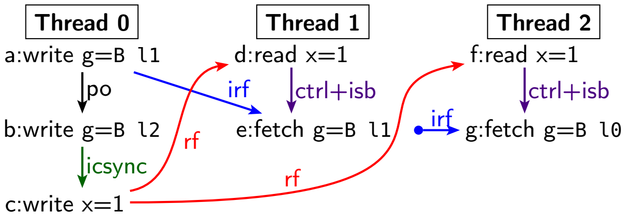

This is not clear in the existing architecture text. It is a case where the architecture design is not very constrained. On the one hand, it has not been observed, and it is thought unlikely that hardware will ever exhibit this behaviour: it would require keeping multiple writes in the coherent part of the data caches, rather than a single dirty line, which would require more complex cache coherence protocols. On the other hand, there does not seem to be any benefit to software from forbidding it. Arm therefore prefer the choice that gives a simpler and weaker model (here the two happen to coincide), to make it easier to understand and to provide more flexibility for future microarchitectural optimisations. We therefore design our models to allow the above behaviour.

#### 3.6 Strength of the `DC` Instruction

**Instruction Cache depth** Test CoFF (§3.2) showed that fetches can see "old" writes. In principle, there is no limit to the depth of the instruction-cache hierarchy: there could be many values for a single location cached in the instruction memory for each core, even if the data cache has been cleaned. The test below illustrates this, with Thread 1 able to see all three values for `g`.

MP.RF+dc+ctrl-isb-isb AArch64

Initial state: `0:W0="B l1", 0:X2=g, 0:W1="B l2", 0:X3=1, 0:X4=x, [x]=0, 1:X4=x`

| Thread 0 | Thread 1 | Common |
|---|---|---|
| `STR W0,[X2]` | `LDR X0, [X4]` | `g: B l0` |
| `STR W1,[X2]` | `CBNZ X0, l` | `l2:MOV X10,#3` |
| `DSB ISH` | `l:`**`ISB`** | `RET` |
| **`DC`** `CVAU,X2` | `BL g` | `l1:MOV X10,#2` |
| `DSB ISH` | `MOV X1,X10` | `RET` |
| `STR X3,[X4]` | **`ISB`** | `l0:MOV X10,#1` |
| | `BL g` | `RET` |
| | `MOV X2,X10` | |
| | **`ISB`** | |
| | `BL g` | |
| | `MOV X3,X10` | |

Allowed: `1:X0=1, 1:X1=3, 1:X2=2, 1:X3=1`

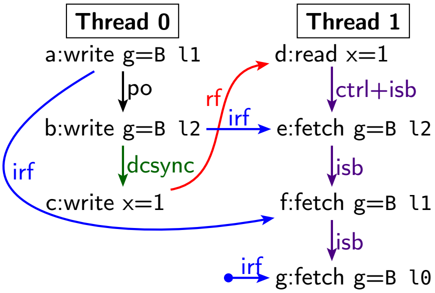

This is similar to the preceding FOW case: it is thought unlikely that hardware will exhibit this in practice, but the desire for the simpler and weaker option means the architectural intent is to allow it, and we follow that in our models.

### 4 An Operational Semantics for Instruction Fetch

Previous work on operational models for IBM POWER and Arm "user-mode" concurrency [46,45,22,18,19,37] has shown, surprisingly, that as far as programmer-visible behaviour is concerned, one can abstract from almost all hardware implementation details of data memory (store queues, the cache hierarchy, the cache protocol, etc.). For ARMv8-A, following their 2018 shift to a multicopy-atomic architecture, one can do so completely: the *Flat* model of [37] has a shared flat memory, with a per-thread out-of-order thread subsystem, modelling pipeline effects, responsible for all observable relaxed behaviour. For instruction-fetch, it is no longer possible to abstract completely from the data and instruction cache hierarchy, but we can still abstract from much of it.

**The Flat Model** is a small-step operational semantics for multi-copy atomic ARMv8-A, including the relaxed behaviours of loads and stores [37]. Its states are abstract machine states consisting of a tree of instructions for each thread, and a flat memory subsystem shared by all threads. Each instruction in each thread corresponds to a sequence of transitions, with some guards and a potential effect on the shared memory state. The Flat model is made executable in our RMEM tool, which can exhaustively interleave transitions to enumerate all the possible behaviours. The tree of instructions for each thread models out-of-order and speculative execution explicitly. Below we show an example for a thread that is executing 10 instruction instances. Some (grey) are finished, no longer subject to restart; others (pink) have run some but perhaps not all of their instruction semantics; instructions are not necessarily atomic. Those with multiple children are branch instructions with multiple potential successors speculated simultaneously.

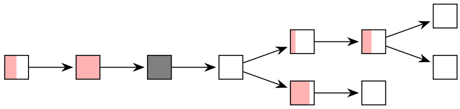

For each state, the model defines the set of allowed transitions, each of which steps to a new machine state. Transitions correspond to steps of single instructions, and individual instructions may give rise to many. Example transitions include Register Write, Propagate Write to Memory, etc.

**iFlat Extension** Originally, Flat had a fixed instruction memory, with a single transition that can speculate the address of any program-order successor of any instruction in flight, fetch it from the fixed instruction memory, and decode it. We now remove that fixed instruction memory, so that instructions can be fetched from data writes, and add the additional structures as shown on the right. These are all of unbounded size, as is appropriate for an architecture definition.

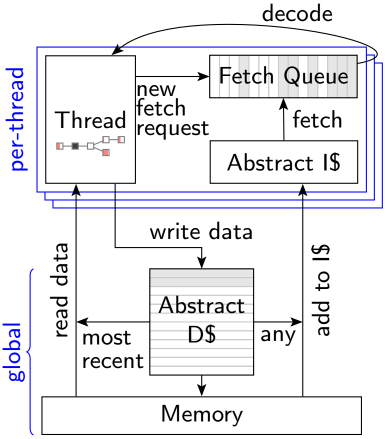

**Fetch Queues (per-thread)** These are ordered buffers of pre-fetched entries, waiting to be decoded and begin execution. Entries are either a fetched 32-bit opcode, or an unfetched request. The fetch queues allow the model to speculate and pre-fetch many instructions ahead of where the thread is currently executing. The model's fetch queues abstract from multiple real-hardware structures: instruction queues, line-fill buffers, loop buffers, and slots objects. We keep a close relation to this underlying microarchitecture by allowing out-of-order fetches, but we believe this is not experimentally observable on real hardware.

**Abstract Instruction Cches (per-thread)** These are just sets of writes. When the fetch queue requests a new entry, it gets satisfied from the instruction cache, either immediately (a *hit*) or at some later point in time (a *miss*). The instruction cache can contain many possible writes for each location (§3.6), and it can be spontaneously updated with new writes in the system at any time ([9, B2.4.4]). To manage `IC` instructions, each thread keeps a list of addresses yet to be invalidated by in-flight `IC`s.

**Data Cache (global)** Above the single shared flat memory for the entire system, which sufficed for the multi-copy-atomic ARMv8-A data memory, we insert a shared buffer which is just a list of writes; abstracting from the many possible coherent data cache hierarchies. Data reads must be coherent, reading from the most recent write to the same address in the buffer, but instruction fetches are allowed to read from any such write in the buffer (§3.2).

**Transitions** To accommodate instruction fetch and cache maintenance, we introduce new transitions: Fetch Request, Fetch Instruction, Fetch Instruction (Unpredictable), Fetch Instruction (B.cond), Decode Instruction, Begin IC, Propagate IC to Thread, Complete IC, Perform DC, and Update Instruction Cache. We also have to modify some Flat transitions: Commit ISB, Wait for DSB, Commit DSB, Propagate Memory Write, and Satisfy Read from Memory. These transitions define the lifecycle of each instruction: a request gets issued for the fetch, then at some later point the fetch gets satisfied from the instruction cache, the instruction is then decoded (in program-order) and then handed to the existing semantics to be executed. To give a flavour, we show just one, the *Propagate IC to Thread* transition, which is responsible for invalidation of the abstract instruction caches. This is a prose rendering of the rule in our executable mathematical model, which is expressed in the typed functional subset of Lem [32].

**Propagate IC to Thread** An instruction *i* (with ID *iiid*) in state WAIT_IC*(address, state_cont)* can do the relevant invalidate for any thread *tid'*, modifying that thread's instruction cache and fetch queue, if there exists a pending entry *(iiid, address)* in that thread's *ic_writes*. Action:

1. for any entry in the fetch queue for thread *tid*, whose *program_loc* is in the same minimum-size instruction cache line as *address*, and is in FETCHED(_) state, set it to the UNFETCHED state;
2. for the instruction cache of thread *tid*, remove any write-slices which are in the same instruction cache line of minimum size as *address*.

This rule can be found under the same name in the full prose description, and in the `handle_ic_ivau` and `flat_propagate_cache_maintenance` functions in `machineDefThreadSubsystem.lem` and `machineDefFlatStorageSubsystem.lem` in the executable mathematics. Cache maintenance operations work over entire cache lines, not individual addresses. Each address is associated with at least one cache line for the data (and unified) caches, and one for the instruction caches. The cache line of minimum size is the (architected) smallest possible cache line for each of these.

**Example** This model correctly explains all the behaviours of §3. We illustrate this by revisiting the cache synchronization explanation of §2, which can now be re-interpreted w.r.t. our precise model, and using this to explain the thread migration case of §3.3. Given `DC Xn; DSB; IC Xn; DSB` we can use this model to give meaning to it (omitting uninteresting transitions): First the `DC CVAU` causes a **Perform DC** transition. This pushes any write that might have been in the abstract data cache into memory. Now the first `DSB`'s **Commit DSB** can be taken, allowing **Begin IC** to happen. This creates entries for each thread, which are discharged by each **Propagate IC to Thread** (see above). Once all entries are invalidated, a **Complete IC** can happen. Now, if any thread decodes an instruction for that address, it must have been fetched from the write the `DC` pushed, or something coherence-after it. If the software thread performing this sequence is interrupted and migrated (by the OS) to a different hardware thread, then, so long as the OS includes the `DSB` to maintain the thread-local DC ordering, the `DC` will push the write in an identical way, since it only affects the global abstract data cache. The `IC` transitions can all be taken, and the sequence continues as before, just on a new hardware thread. So when the second `DSB` finishes, and the final **Commit DSB** transitions is taken, the effect of the full sequence will be seen system-wide even if the thread was migrated.

### 5 An Axiomatic Semantics for Instruction Fetch

Based on the operational model, we develop an axiomatic semantics, as an extension of the ARMv8 axiomatic reference model [15,37]. Since that does not have mixed-size support, we do not model the concurrent modification of conditional branches (§3.1), as this would require mixed-size machinery. The existing axiomatic model is a predicate on *candidate executions*, hypothetical complete executions of the given program that satisfy some basic well-formedness conditions, defining the set of *valid* executions to be those satisfying its axioms. Each candidate execution abstractly captures a particular concrete execution of the program in terms of events and relations over them. This model is expressed in the herd language [8,6,4]. The events of these executions are memory reads (the set `R`), memory writes (`W`), and memory barrier/fence events (`F`). The relations are: *program order* (`po`), capturing the sequencing of events by the same thread in the execution's control-flow unfolding; *reads-from* (`rf`), relating a write event $w$ with any read event $r$ that reads from it; the *coherence order* (`co`), recording the execution's sequencing of same-address writes in memory; and *read-modify-write* (`rmw`), capturing which load/store exclusive instructions form a successful exclusive *pair* in the execution. The derived relation *from-reads* $\mathtt{fr} = \mathtt{rf}^{-1}\mathtt{;co}$ relates a read $r$ with a write $w'$ if $r$ reads from a write $w$ coherence before $w'$. In addition, candidate executions also have relations capturing dependencies between events: address (`addr`), data (`data`), and control dependencies (`ctrl`). The relation `loc` relates any two read/write events that are to the same memory address. The model also has relations suffixed "i" and "e": `rfi`/`rfe`, `coi`/`coe`, `fri`/`fre`. These are the restrictions of the relations `rf`, `co`, and `fr`, to same-thread/"internal" event pairs or different-thread/"external" event pairs. The model is defined in relational algebra. In herd, `R;S` stands for sequential composition of relations `R`

and S, $\texttt{R}^{-1}$ for the inverse of relation R, R|S and R&S for the union and intersection of R and S, and [A];R;[B] for the restriction of R to the domain A and range B.

Handling instruction fetch requires extending the notion of candidate execution. We add new events: an *instruction-fetch* (IF) event for each executed instruction; a DC event for each DC CVAU instruction; an IC event for each IC IVAU and IC IALLU instruction. We replace po with *fetch-program-order* (fpo) which orders the IF event of an instruction before any program-order later IF events. We add a relation *same-cache-line* (scl), relating reads, writes, fetches, DC and IC events to addresses in the same cache line. We add an acyclic transitively closed relation wco, which extends co with orderings for cache maintenance (DC or IC) events: it includes an ordering $(e, e')$ or $(e', e)$ for any cache maintenance event $e$ and same-cache-line event $e'$ if $e'$ is a write or another cache maintenance event; where co = ([W];wco;[W]) & loc. The loc, addr, and ctrl are all extended to include DC and IC events. We add a *fetch-to-execute* relation (fe), relating an IF event to any event generated by the execution of that instruction; and an *instruction-read-from* relation (irf), which relates a write to any IF event that fetches from it. Finally, we add a boolean *constrained-unpredictable* (CU) to detect badly behaved programs. Now we derive the following relations: the standard po relation, as po = $\texttt{fe}^{-1}$;fpo;fe (two events $e$ and $e'$ are po-related if their fetch-events are fpo-related); and *instruction-from-reads* (ifr), the analogue of fr for instruction fetches, relating a fetch to all writes coherence-after the one it fetched from: ifr = $\texttt{irf}^{-1}$;co.

We then make two semantics-preserving rewrites of the existing model to make adding instruction fetches easier (described in the appendix); and make the following changes and additions to the model. The full model is shown in Figure 1, with comments pointing to the relevant locations in the model definition. For lack of space we only describe the main addition, the iseq relation, in detail (including its correspondence with the operational model of §4); for the others we give an overview and refer to the appendix for the full description.

We define the relation iseq, relating some write $w$ to address $x$ to an IC event completing a cache synchronisation sequence (not necessarily on a single thread): $w$ is followed by a same-cache line DC event, which is in turn followed by a same-cache line IC event. In operational model terms, this captures traces that propagated $w$ to memory, subsequently performed a same-cache-line DC, and then began an IC (and eagerly propagated the IC to all threads). In any state after this sequence it is guaranteed that $w$, or a coherence-newer same-address write, is in the instruction cache of all threads: performing the DC has cleared the abstract data cache of writes to $x$, and the subsequent IC has removed old instructions for location $x$ from the instruction caches, so that any subsequent updates to the instruction caches have been with $w$, or co-newer writes. Adding ifr;iseq to the *observed-by* relation (obs) (4) relates an instruction fetch $i$ to location $x$ to an IC $ic$ if: $i$ fetched from a write $w$ to $x$, some write $w'$ to $x$ is coherence-after $w$, and $ic$ completes a cache synchronisation sequence (iseq) starting from $w'$. Then the irreflexive ob axiom requires that $i$ must be ordered-before $ic$ (because it would otherwise have fetched $w'$).We now

```
let iseq = [W];(wco&scl);[DC];   (*1*)
               (wco&scl);[IC]

(* Observed-by *)
let obs = rfe | fr | wco         (*2*)
        | irf | (ifr;iseq)       (*3,4*)

(* Fetch-ordered-before *)
let fob = [IF]; fpo; [IF]        (*5*)
  | [IF]; fe                     (*6*)
  | [ISB]; fe^-1; fpo            (*7*)

(* Dependency-ordered-before *)
let dob = addr | data
  | ctrl; [W]
  | (ctrl | (addr; po)); [ISB]
(*| [ISB]; po; [R] *)            (*8*)
  | addr; po; [W]
  | (addr | data); rfi

(* Atomic-ordered-before *)
let aob = rmw
  | [range(rmw)]; rfi; [A|Q]

(* Barrier-ordered-before *)
let bob = [R|W]; po; [dmb.sy]
  | [dmb.sy]; po; [R|W]
  | [L]; po; [A]
  | [R]; po; [dmb.ld]
  | [dmb.ld]; po; [R|W]
  | [A|Q]; po; [R|W]
  | [W]; po; [dmb.st]
  | [dmb.st]; po; [W]
  | [R|W]; po; [L]
  | [R|W|F|DC|IC]; po; [dsb.ish]  (*9*)
  | [dsb.ish]; po; [R|W|F|DC|IC]  (*10*)
  | [dmb.sy]; po; [DC]            (*11*)

(* Cache-op-ordered-before *)
let cob = [R|W]; (po&scl); [DC]   (*12*)
  | [DC]; (po&scl); [DC]          (*13*)

(* Ordered-before *)
let ob = (obs|fob|dob|aob|bob|cob)+

(* Internal visibility requirement *)
acyclic (po-loc|fr|co|rf) as internal

(* External visibility requirement *)
irreflexive ob as external

(* Atomic *)
empty rmw & (fre; coe) as atomic

(* Constrained unpredictable *)
let cff = ([W];loc;[IF]) \        (*14*)
             ob^-1 \ (co;iseq;ob)
cff_bad cff ≡ CU                  (*15*)
```

**Fig. 1.** Axiomatic model briefly overview other changes made to the axiomatic model and their intuition. We include `irf` in `obs` `(3)`: for an instruction to be fetched from a write, the write has to have been done before. We add a relation *fetch-ordered-before* (`fob`) `(5-7)`, which is included in *ordered-before*. The relation `fob` includes `fpo` and `fe`; including `fpo` `(5)` requires fetches to be ordered according to their position in the control-flow unfolding of the execution. and including the `fe` (*fetch-to-execute*) relation `(6)` captures the idea that an instruction must be fetched before it can execute; fetches program-order-after an ISB happen after the ISB (or else are restarted) `(7)`. For `DSB ISH` instructions the edge `[R|W|F|DC|IC];po;[dsb.ish]` is included in `ob` `(9)`: `DSB ISH`s are ordered with all program-order-preceding non-fetch events. Symmetrically, all non-`IF` events are ordered after program-order-preceding `dsb.ish` events `(10)`. `DC`s wait for preceding `dmb.sy` events `(11)`. We include the relation *cache-op-ordered-before* (`cob`) in `ob`. This relation orders `DC` instructions with program-order previous reads/writes and other `DC`s to the same cache line `(12,13)`.

Finally, *could-fetch-from* (`cff`) `(14)` captures, for each fetch $i$, the writes it could have fetched from (including the one it did fetch from), which we use to define the *constrained unpredictable* axiom `cff_bad` (not given) `(15)`.

## 6 Validation

To gain confidence in the presented models we validated the models against the Arm architectural intent, against each other, and against real hardware.

**Validation against the Architecture** To ensure our models correctly captured the architectural intent we engaged in detailed discussions with Arm, including the Arm chief architect. These involved inventing litmus tests (including, those described in §3 and many others) and discussing what the architecture should allow in each case.

**Validating against hardware** To run instruction-fetch tests on hardware, we extended the litmus tool [7]. The most significant extension consists in handling code that can be modified, and thus has to be restored between experiments. To that end, code copies are executed, those copies reside in mmap'd memory with (execute permission granted. Copies are made from "master" copies, in effect C functions whose contents basically consist of gcc extended inline assembly. Of course, such code has to be position independent, and explicit code addresses in test initialisation sections (such as in `0:X1=l` in the test of §3.1) are specific to each copy. All the cache handling instructions used in our experiments are all allowed to execute at exception level 0 (user-mode), and therefore no additional privilege is needed to run the tests.

To automatically generate families of interesting instruction-fetch tests, we extended the diy test generation tool [3] to support instruction-fetch reads-from (`irf`) and instruction-fetch from-reads (`ifr`) edges, in both internal (same-thread) and external (inter-thread) forms, and the `cachesync` edge. We used this to generate 1456 tests involving those edges together with `po`, `rf`, `fr`, `addr`, `ctrl`, `ctrlisb`, and `dmb.sy`. diy does not currently support bare `DC` or `IC` instructions, locations which are both fetched and read from, or repeated fetches from the same location.

We then ran the diy-generated test suite on a range of hardware implementations, to collect a substantial sample of actual hardware behaviour.

**Correspondence between the models** We experimentally test the equivalence of the operational and axiomatic models on the above hand-written and diy-generated tests, checking that the models give the same sets of allowed final states, and that these are consistent with the hardware observations.

**Making the models executable as a test oracle** To make the operational model executable as a test oracle, capable of computing the set of all allowed executions of a litmus test, we must be able to *exhaustively enumerate* all possible traces. For the model as presented, doing this naively is infeasible: for each instruction it is theoretically possible to speculate any of the $2^{64}$ addresses as potential next address, and the interleaving of the new fetch transitions with others leads to an additional combinatorial explosion.

We address these with two new optimisations. First, we extend the fixed-point optimisation in RMEM (incrementally computing the set of possible branch targets) [37] to keep track not only of indirect branches but also the successors of every program location, and only allow speculating from this set of successors. Additionally, we track during a test which locations were both fetched and modified during the test, and eagerly take fetch and decode transitions for all other locations. As before, the search then runs until the set of branch targets *and* the set of modified program-locations reaches a fixed point. We also take some of the transitions eagerly to reduce the search space, in cases where this cannot remove behaviour: **Wait for IC**, **Complete IC**, **Fetch Request**, and **Update Instruction Cache**.

**Making the axiomatic model executable as a test oracle** The axiomatic model is expressed in a herd-like form, but the herd tool does not support instruction fetch and cache maintenance instructions. To make the model executable as a test oracle, we built a new tool that takes litmus tests and uses a Sail [11] definition of a fragment of the ARMv8-A ISA to generate SMT problems for the model. Using the Sail instruction semantics, we generate a Sail program that corresponds to each thread within a litmus test. The tool then partially evaluates these programs using the concrete values for addresses and registers specified in the litmus file, while allowing memory values and arbitrary addresses to remain symbolic. Using a Sail to SMT-LIB backend, these are translated into SMT definitions that include all possible behaviours of each thread as satisfiable solutions. The rules for the axiomatic model are then applied as assertions restricting the possible behaviours to just those allowed by the axiomatic model. The tool also derives the `addr` and `data` relations, using the syntactic dependencies within the instruction semantics to derive the syntactic dependencies between instructions.

For litmus tests, where we can know up-front which instructions may be modified, we would like to avoid generating `IF` events for instructions that cannot be modified. If we naively removed certain `IF` events, however, we would break the correspondence between `po` and $\texttt{fe}^{-1}\texttt{;fpo;fe}$. This can be worked around by ensuring that every modifiable instruction generates an event which appears in `po`, allowing `fpo` between the modifiable instructions to instead be derived as $\texttt{fe;po;fe}^{-1}$. Branches emit a special branch address announce event for this purpose, which is also used to derive the `ctrl` relation. The `fpo` relation can then be modified, replacing $\texttt{[ISB];fe}^{-1}\texttt{;fpo}$ with $\texttt{[ISB];po;fe}^{-1}$ and adding `[ISB];po`. The second change ensures that all the transitive edges generated by $\texttt{[ISB];fe}^{-1}\texttt{;fpo}$ followed by `[IF];fe` remain with `fob` and hence `ob`.

A limitation of this approach is it cannot support cases where two threads both attempt to execute the same possibly-modified instruction, as in the SM.F+ic and FOW tests.

**Validation results** First, to check for regressions, we ran the operational model on all the 8950 non-mixed-size tests used for developing the original Flat model (without instruction fetch or cache maintenance). The results are identical, except for 23 tests which did not terminate within two hours. We used a 160 hardware-thread POWER9 server to run the tests.

We have also run the axiomatic model on the 90 basic two-thread tests that do not use Arm release/acquire instructions (not supported by the ISA semantics used for this); the results are all as they should be. This takes around 30 minutes on 8 cores of a Xeon Gold 6140.

Then, for the key handwritten tests mentioned in this paper, together with some others (that have also been discussed with Arm), we ran them on various hardware implementations and in the operational and axiomatic models. The models' results are identical to the Arm architectural intent in all cases, except for two tests which are not currently supported by the axiomatic checker.

| Test | Arm intent | op. model | ax. model | hardware obs. |
|---|---|---|---|---|
| CoFF | allow | = | = | 42.6k/13G |
| CoFR | forbid | = | = | 0/13G |
| CoRF+ctrl-isb | allow | = | = | 3.02G/13G |
| SM | allow | = | = | 25.8G/25.9G |
| SM+cachesync-isb | forbid | = | = | 0/25.9G |
| MP.RF+dmb+ctrl-isb | allow | = | = | 480M/6.36G |
| MP.RF+cachesync+ctrl-isb | forbid | = | = | 0/13G |
| MP.FR+dmb+fpo-fe | forbid | = | = | 0/13G |
| MP.FF+dmb+fpo | allow | = | = | 447M/13G |
| MP.FF+cachesync+fpo | forbid | = | = | [F] 2.3k/13G |
| ISA2.F+dc+ic+ctrl-isb | forbid | = | = | 0/6.98G |
| SM.F+ic | allow | = | unsupported | [U] 0/12.9G |
| FOW | allow | = | unsupported | [U] 0/7G |
| MP.RF+dc+ctrl-isb-isb | allow | = | = | [U] 0/12.94G |
| MP.R.RF+addr-cachesync+dmb+ctrl-isb | forbid | = | = | 0/6.97G |
| MP.RF+dmb+addr-cachesync | allow | = | = | [U] 0/6.34G |

[The hardware observations are the sum of testing seven devices: a Snapdragon 810 (4x Arm A53 + 4x Arm A57 cores), Tegra K1 (2x NVIDIA Denver cores), Snapdragon 820 (4x Qualcomm Kryo cores), Exynos 8895 (4x Arm A53 + 4x Samsung Mongoose 2 cores), Snapdragon 425 (4x Arm A53), Amlogic 905 (4x Arm A53 cores), and Amlogic 922X (4x Arm A73 + 2x Arm A53 cores). U: allowed but unobserved. F: forbidden but observed.]

Our testing revealed a hardware bug in a Snapdragon 820 (4 Qualcomm Kryo cores). A version of the first cross-thread synchronisation test of §3.3 but with the full cache synchronisation (MP.RF+cachesync+ctrl-isb) exhibited an illegal outcome in 84/1.1G runs (not shown in the table), which we have reported. We have also seen an anomaly for MP.FF+cachesync+fpo, currently under investigation by Arm. Apart from these, the hardware observations are all allowed by the models. As usual, specific hardware implementations are sometimes stronger.

Finally, we ran the 1456 new instruction-fetch diy tests on a variety of hardware, for around 10M iterations each, and in the operational model. The model is sound with respect to the observed hardware behaviour except for that same Snapdragon 820 device.

### 7 Related Work

To the best of our knowledge, no previous work establishes well-validated rigorous semantics for any systems aspects, of any current production architecture, in a realistic concurrent setting.

The closest is Raad et al.'s work on non-volatile memory, which models the required cache maintenance for persistent storage in ARMv8-A [39], as an extension to the ARMv8-A axiomatic model, and for Intel x86 [38] as an operational model, but neither are validated against hardware. In the sequential case, Myreen's JIT compiler verification [33] models x86 icache behaviour with an abstract cache that can be arbitrarily updated, cleared on a `jmp`. For address translation, the authoritative Arm-internal ASL model [40,41,42], and Sail model derived from it [11] cover this, and other features sufficient to boot an OS (Linux), as do the handwritten Sail models for RISC-V (Linux and FreeBSD) and MIPS/CHERI-MIPS (FreeBSD, CheriBSD), but without any cache effects. Goel et al. [21,20] describe an ACL2 model for much of x86 that covers address translation; and the Forvis [34] and RISCV-PLV [14] Haskell RISC-V ISA models are also complete enough to boot Linux. Syeda and Klein [49,50] provide an somewhat idealised model for ARMv7 address translation and TLB maintenance. Komodo [16] uses a handwritten model for a small part of ARMv7, as do Guanciale et al. [25,12]. Romanescu et al. [44,43] do discuss address translation in the concurrent setting, but with respect to idealised models. Lustig et al. [30] describe a concurrent model for address translation based on the Intel Sandy Bridge microarchitecture, combined with a synopsis of some of the relevant Linux code, but not an architectural semantics for machine-code programs.

### 8 Conclusion

The mainstream architectures are the most important programming languages used in practice, and their systems aspects are fundamental to the security (or lack thereof) of our computing infrastructure. We have established a robust semantics for one of those systems aspects, soundly abstracting the hardware complexities to a manageable model that captures the architectural intent. This enables future work on reasoning, model-checking, and verification for real systems code.

**Acknowledgements** This work would not have been possible without generous technical assistance from Arm. We thank Richard Grisenthwaite, Will Deacon, Ian Caulfield, and Dave Martin for this. We also thank Hans Boehm, Stephen Kell, Jaroslav Ševčík, Ben Titzer, and Andrew Turner, for discussions of how instruction cache maintenance is used in practice, and Alastair Reid for comments on a draft. This work was partially supported by EPSRC grant EP/K008528/1 (REMS), ERC Advanced Grant 789108 (ELVER), an ARM iCASE award, and ARM donation funding. This work is part of the CIFV project sponsored by the Defense Advanced Research Projects Agency (DARPA) and the Air Force Research Laboratory (AFRL), under contract FA8650-18-C-7809. The views, opinions, and/or findings contained in this paper are those of the authors and should not be interpreted as representing the official views or policies, either expressed or implied, of the Department of Defense or the U.S. Government.
